# Unraveling the Role of Perivascular Macrophages in Alzheimer's Disease: Insights from the Crosstalk between Immunometabolism and Ferroptosis

**DOI:** 10.2174/011570159X417328250908080404

**Published:** 2025-09-18

**Authors:** Xiaolei Miao, Wei Yue, Jinxu Wang, Jiahui Chen, Lei Qiu, Halisa Paerhati, Qin Zhou, Pengyi Li, Anshi Wu, Minhao Zhang

**Affiliations:** 1 Department of Anesthesiology, Beijing Chao-Yang Hospital, Capital Medical University, Beijing, 100020, China;; 2 Jiangsu Province Key Laboratory of Anesthesiology, Xuzhou Medical University, Xuzhou, Jiangsu Province, 221004, China;; 3 Department of Anesthesiology, Women’s Hospital of Nanjing Medical University, Nanjing Maternity and Child Health Care Hospital, Nanjing, Jiangsu Province, 210009, China;; 4 Department of Anesthesiology, Jiangsu Cancer Hospital and Jiangsu Institute of Cancer Research and the Affiliated Cancer Hospital of Nanjing Medical University, Nanjing, Jiangsu Province, 210009, China

**Keywords:** Alzheimer’s disease, perivascular macrophages, single-cell RNA sequencing, mendelian randomization, immune infiltration, ferroptosis

## Abstract

**Introduction:**

Recent evidence increasingly supports a potential role of Perivascular Macrophages (PVMs), a unique subpopulation of brain immune cells, in the pathogenesis of Alzheimer’s disease (AD). Strategically positioned at the brain-vasculature interface, PVMs sense the redox status, modulate immunity, and potentially influence ferroptosis—an iron-dependent form of regulated cell death increasingly implicated in AD. However, whether the involvement of PVMs in AD pathology specifically entails mechanisms related to the crosstalk between immunometabolism and ferroptosis, and the precise molecular pathways linking PVMs, immunometabolism, and ferroptosis to AD, remains unclear.

**Methods:**

We first obtained single-cell RNA sequencing data of PVMs from AD patients and control subjects *via * the GEO database, identified Differentially Expressed Genes (DEGs), and applied Mendelian Randomization (MR), with robustness validated *via * leave-one-out analysis to pinpoint key genes among the DEGs with causal relevance to AD. Next, we identified ferroptosis-related genes within these key genes and examined their associations with immune cell infiltration and immunometabolic signaling pathways, while also predicting their regulatory transcription factors to inform potential therapeutic strategies.

**Results and Discussion:**

We identified 149 DEGs in PVMs between AD and control groups, which were primarily enriched in immune and metabolic pathways. MR analysis established eight genes ( *ACSL1* , *SPATA6* , *RAB31* , *NIBAN1* , *HDAC4* , *GRAMD1B* , *GCC2* , and *DENND3* ) as causally and negatively associated with AD risk (IVW analysis identified all *P* < 0.05, with robustness confirmed by leave-one-out analysis), with *ACSL1* being recognized as a known ferroptosis driver. Immune cell infiltration analysis revealed significant differences in monocyte and neutrophil proportions in AD, with *DENND3* identified as the sole gene significantly correlated with monocyte abundance. The Key genes demonstrated distinct associations with immunometabolic pathways: *GRAMD1B* expression was positively associated with PI3K/AKT/mTOR signaling, whereas both *NIBAN1* and *SPATA6* showed enrichment in cells with high Notch signaling activity. *ACSL1* exhibited robust associations with multiple pathways implicated in ferroptosis, including the IL-6/JAK/STAT3, interferon-γ, TGF-β, bile acid metabolism, and cholesterol homeostasis pathways, suggesting potential mechanisms that mediate the crosstalk between immunometabolism and ferroptosis. Transcription factor analysis highlighted shared regulation by CEBPD and the SP1/2/3/4 family, indicating convergent transcriptional control of these genes.

**Conclusion:**

This study identifies eight key genes in PVMs that may protect against AD through mechanisms involving the interplay between immunometabolism and ferroptosis. Our findings provide novel insights into the function of PVMs in AD pathophysiology and suggest potential therapeutic targets for this devastating neurodegenerative disease.

## 1 INTRODUCTION

As the elderly population continues to grow, the prevalence of age-related diseases, including dementia, has been increasing [1]. Alzheimer’s disease (AD), the most prevalent form of dementia, accounts for approximately 60-70% of these cases [2]. AD is primarily characterized by early memory impairment and cognitive decline, which may eventually extend to affect behavior, language, visuospatial orientation, and motor functions. Despite various molecular-level attempts to elucidate the fundamental biological mechanisms contributing to this disease [3, 4], the primary mechanisms underlying its onset remain unclear, hindering the discovery of disease-modifying therapies [5].

 Compelling evidence suggests that immune system dysfunction plays a role in the progression and development of AD [6, 7]. The central nervous system (CNS)-associated innate immune system comprises two types of resident brain immune cells: microglia in the brain parenchyma and border-associated macrophages within the meninges, choroid, and perivascular spaces [8, 9]. Most studies in this field have focused on microglia, as these cells play a central role in immune regulation within the CNS [7, 10, 11]. Little information is available on border-associated macrophages, such as perivascular macrophages (PVMs).

PVMs are predominantly found near arteries and veins within the perivascular space. Physiologically, these cells contribute to brain homeostasis by offering both structural and functional support. Their roles include maintaining blood-brain barrier integrity, facilitating lymphatic drainage, performing immune functions such as phagocytosis and antigen presentation, and regulating lipid metabolism [12, 13]. Increasing evidence also suggests they have a role in the etiopathogenesis of several disorders of the brain, including AD [14-16]. PVMs might perform a dual function in AD, either clearing amyloid-beta (Aβ) or metabolic waste from the brain, or secreting pro-inflammatory cytokines that can impair the neurovascular unit [17]. However, the exact process by which PVMs are involved in the etiology of AD remains unknown.

Emerging evidence further suggests that PVMs may be involved in adverse complications associated with medications recently approved by the Food and Drug Administration (FDA) for treatment of AD, making it increasingly important to understand their role in the pathophysiology of this disease. Since pathophysiological changes in AD predominantly involve the accumulation of Aβ protein, anti-Aβ antibodies, such as donanemab, lecanemab, and aducanumab, have recently been approved by the FDA for the treatment of AD [1, 18, 19], representing a major breakthrough in AD therapy. Despite this advancement, unwanted effects of these therapies have been reported [20, 21]. The underlying mechanisms of these adverse events remain poorly understood; nonetheless, recent findings suggest a potential involvement of PVMs. Postmortem examination of a patient treated with lecanemab, who later succumbed to a brain hemorrhage, revealed the presence of Aβ-phagocytosing PVMs, along with cerebral vasculitis [22]. Similarly, AD mice treated with anti-Aβ antibodies exhibited accumulation of PVMs at sites of vascular amyloid immune complexes [23]. Therefore, elucidating the specific role of PVMs in AD may offer valuable insights for the development of disease-modifying therapies.

Moreover, research has also confirmed that ferroptosis is a significant mechanism underlying both intracerebral hemorrhage and subarachnoid hemorrhage [24, 25]. Ferroptosis, an iron-dependent form of regulated cell death characterized by redox imbalance and lipid peroxidation, is considered a potential contributor to the pathophysiology of AD and has attracted widespread research attention in recent years [26]. While studies have established a relationship between microglia and ferroptosis [27], the connection between PVMs and ferroptosis remains unclear. Given their strategic positioning at the neurovascular interface, PVMs are uniquely situated to monitor cerebral redox status and regulate immune and metabolic homeostasis—processes fundamentally linked to ferroptotic activation—making them plausible participants in this pathway. However, whether the involvement of PVMs in AD pathology specifically includes mechanisms of the crosstalk between immunometabolism and ferroptosis remains to be determined. A comprehensive characterization of the function of PVMs in neurodegenerative diseases could lead to significant therapeutic advances, potentially mitigating adverse effects associated with anti-Aβ antibody treatments or facilitating the development of more effective disease-modifying interventions for AD.

By understanding the key genes involved, we can better elucidate the disease’s pathogenesis and potentially identify new therapeutic targets. Genome-Wide Association Studies (GWAS) have identified numerous genetic variants linked to AD, with a predominant expression in microglial cells involved in phagocytosis [28-30]. Nonetheless, few studies have intensively explored the key genes involved in AD in PVMs to date [31].

In recent times, Mendelian Randomization (MR) has emerged as a new method of establishing causal associations between exposures and outcomes [32, 33]. Within this analytical method, scientists employ genetic factors that follow Mendelian inheritance principles and utilize single-nucleotide polymorphisms (SNPs) as instrumental variables (IVs) to make inferences about associations between exposures and clinically relevant outcomes [34]. With MR, scientists can model interactions between SNP-gene expression information and the associations of SNPs with diseases to infer causal associations between exposures and outcomes. An MR study could be carried out using suitably designed and available data from two large-scale GWAS, in which exposures and outcomes can be evaluated from two separate cohorts [35, 36]. Nonetheless, MR has not yet been applied to study the cause-effect relationship between PVMs and AD.

In this study, we first employed single-cell RNA sequencing (scRNA-seq) to identify Differentially Expressed Genes (DEGs) in PVMs between AD and control groups. Then, we conducted MR analysis to identify key genes associated with AD and further identified ferroptosis-related genes (FRGs) among these key genes by querying the FerrDb V2 database. After confirming their robustness through leave-one-out analysis, we examined the relationships between these genes and both Immune Cell Infiltration (ICI) and various signaling pathways—particularly those involved in immunometabolism—to elucidate their potential roles in AD through the lens of the crosstalk between immunometabolism and ferroptosis. Additionally, to guide future therapeutic approaches, we identified the Transcription Factors (TFs) that regulate these genes. Collectively, this research elucidates the role of PVMs in AD from the perspective of the crosstalk between immunometabolism and ferroptosis, suggests new connections between innate immune cells and AD, and provides novel and potential therapeutic targets for this devastating condition.

## 2 MATERIALS AND METHODS

### 2.1 Data Sources

Dataset GSE264648 was downloaded from the Gene Expression Omnibus (GEO; https://www.ncbi.nlm.nih.gov/geo/) database for single-cell analysis [37]. It comprises expression profile information from 49 cases, of which 24 were controls and 25 were classified as disease. In addition, dataset GSE153873 was downloaded for immune-related investigation, with its associated annotation file being GPL18573. This dataset includes expression profiles of 30 patients, comprising 18 controls and 12 patients with the disease.

Exposure data: Expression Quantitative Trait Loci (eQTL) information was derived from the eQTLGen consortium database (https://www.eqtlgen.org), which examines the genetic architecture of blood expression and unravels the genetic basis underlying complex traits [38]. The eQTLGen project is currently in its second phase, focusing on conducting a large-scale genome-wide meta-analysis of blood samples [38].

Outcome data: The outcome summary data (ID: ebi-a-GCST90027158) was obtained from the EBI database (https://gwas.mrcieu.ac.uk/). The GWAS studies included primarily participants of European ancestry.

### 2.2 scRNA-seq Data Processing

The Seurat package was utilized for scRNA-seq data analysis [39]. Cells were filtered based on factors such as the total Unique Molecular Identifier (UMI) count, the number of expressed genes, and the proportion of mitochondrial and ribosomal gene expression. Cells exhibiting high UMI counts and numerous expressed genes were classified as doublets. In contrast, those with elevated mitochondrial and ribosomal gene expression were considered of poor quality, indicating potential apoptosis or degradation into cellular debris. Outliers were defined as data points that were three Median Absolute Deviations (MAD) from the median. Quality control was conducted based on the following criteria in this study: nFeature_RNA >= 200 & percent.mt <= 3*MAD & nFeature_RNA <= 3*MAD & nCount_RNA <= 3*MAD & percent.ribo <= 3*MAD. DoubletFinder (version 2.0.4) was utilized to identify and remove doublets from each sample, thereby completing the quality control process.

Normalization of the data was carried out using the “NormalizeData” function. The “CellCycleScoring” function was applied to calculate the cell-cycle scores, and hypervariable genes were detected using the “FindVariableFeatures” function. The “ScaleData” function was used to adjust for variations in gene expression related to mitochondrial gene expression, ribosomal gene proportions, and cell-cycle differences. Linear dimensionality reduction was performed using “RunPCA”, with principal components selected for further analysis. Harmony was implemented to correct for batch effects, and Uniform Manifold Approximation and Projection (UMAP) was applied for nonlinear dimensionality reduction. The “FindNeighbors” function identified neighboring cells, while “FindClusters” was used to separate the cells into distinct clusters. Cell type annotation was performed by querying databases such as CellMarker and PanglaoDB, along with a review of relevant literature. The average gene expression level for each cell type was calculated using the “AverageExpression” function. DEGs were identified using the “FindMarker” function, applying the following filters: |avg_log_2_FC| > 0.25 and *P* < 0.05.

### 2.3 Analysis of GO and KEGG Functions

The DEGs were annotated functionally using the Metascape database (www.metascape.org) [40]. Gene Ontology (GO) and Kyoto Encyclopedia of Genes and Genomes (KEGG) analyses were performed to determine the biological functions and pathways of these genes involved in disease progression [41]. A minimum overlap of ≥ 3 and *P* ≤ 0.01 was considered significant.

### 2.4 MR Analysis

 The EBI database provides comprehensive summary statistics. Outcome IDs extracted from the GWAS summary data were filtered through the EBI database. SNPs meeting the significance threshold (*P* < 1e-8) were selected as potential IVs, and the Linkage Disequilibrium (LD) between these SNPs was evaluated. Only SNPs with an R2 value lower than 0.001, based on a clumping window size of 10,000 kb, were retained for further analysis. Weak IVs were excluded if their F-statistic was greater than 10 [42-44]. MR analysis was performed using Inverse Variance Weighting (IVW), MR Egger, weighted median, and weighted mode methods to assess the causal relationship. The weighted mode offers superior detection capacity, lower bias, and reduced type I error rates. In cases where only a single statistical method was applicable, the Wald ratio was used to assess the overall effect of all cis-regions and selected cross-regions on AD in whole blood specimens.

 A Mendelian heterogeneity test was conducted to assess statistical heterogeneity among the studied SNPs. This involved calculating the weighted sum of squares of the effect sizes, along with their corresponding standard errors for each SNP, yielding a Q value. If the *P*-value associated with the Q value exceeded 0.05, we concluded that there was no heterogeneity.

We employed a leave-one-out analysis as a sensitivity measure to assess the influence of individual genetic variants on the risk of AD. This approach involves systematically removing each SNP one at a time and recalculating the overall effect size of the remaining SNPs. By excluding variants that disproportionately contribute to the overall estimate, we obtained a new point estimate along with a 95% Confidence Interval (CI) for each iteration. By comparing the estimates obtained after removing individual SNPs to those obtained with all SNPs included, we evaluated the impact of each SNP’s removal on the overall results, thereby assessing the robustness of the analysis.

### 2.5 ICI Analysis

The CIBERSORT method utilizes support vector regression to perform deconvolution analysis on the expression matrix of immune cell subtypes. The method includes 547 biomarkers that distinguish 22 human immune cell phenotypes. The correlation between gene expression levels and immune cell content was also assessed.

Using the ‘Gene’ module of TISIDB (http://cis.hku.hk/TISIDB/), we extracted the pre-computed Spearman correlations between our eight key genes and the infiltration scores of various immune-cell subsets and immunomodulators. Correlation tables were downloaded, imported into R 4.3.1, and two-tailed *P* values were recalculated from the original TCGA log2-TPM matrices to ensure reproducibility. The *P* values were FDR-adjusted using the Benjamini-Hochberg method (threshold < 0.05). Significant associations were visualized using ggplot2.

### 2.6 Regulatory Network Analysis of Key Genes

The package “RcisTarget” was used to predict TFs associated with the key genes identified [45]. All calculations performed with RcisTarget were based on the presence of specific motifs. The Normalized Enrichment Score (NES) of a motif depends on the total number of motifs present in the database. In addition to the motifs already annotated in the source data, further annotations were derived based on motif similarity and gene sequences. To assess the overexpression of each motif in a gene set, we initially calculated the Area Under the Curve (AUC) for each motif-motif set pair. This was done using recovery curve calculations of the gene set *versus* the order of motifs.

### 2.7 Statistical Analysis

MR analysis was performed using R software (version 4.3.0). *P* < 0.05 was considered statistically significant.

## 3 RESULTS

### 3.1 Identification of 149 DEGs and Enrichment Analysis Results

To identify key genes in PVMs involved in AD pathogenesis, we first conducted scRNA-seq analysis using the expression profile obtained from the GEO database. After excluding low-quality cells, 161,638 cells were retained (Figs. **S1A** and **B**). Gene expression levels across samples, along with the top 10 genes with the highest standardized variance, are shown in Fig. (**S1C**). The data processing included homogenization, principal component analysis, and harmony analyses (Figs. **S1D-F**). UMAP analysis was employed to visualize the positional relationships between clusters, revealing 18 distinct cell clusters (Fig. **S2A**). These clusters were further classified into eight major cell groups: oligodendrocytes, astrocytes, excitatory neurons, oligodendrocyte precursor cells, PVMs, inhibitory neurons, vascular leptomeningeal cells, and endothelial cells (Fig. **S2B**). Bubble plots displaying classical markers for these eight cell groups, as well as the proportions of different cell types within the groups (Figs. **S2C** and **S2D**), respectively. These findings suggest that changes in the distribution and proportions of various cell types are involved in AD pathogenesis.

**Fig. (1):**
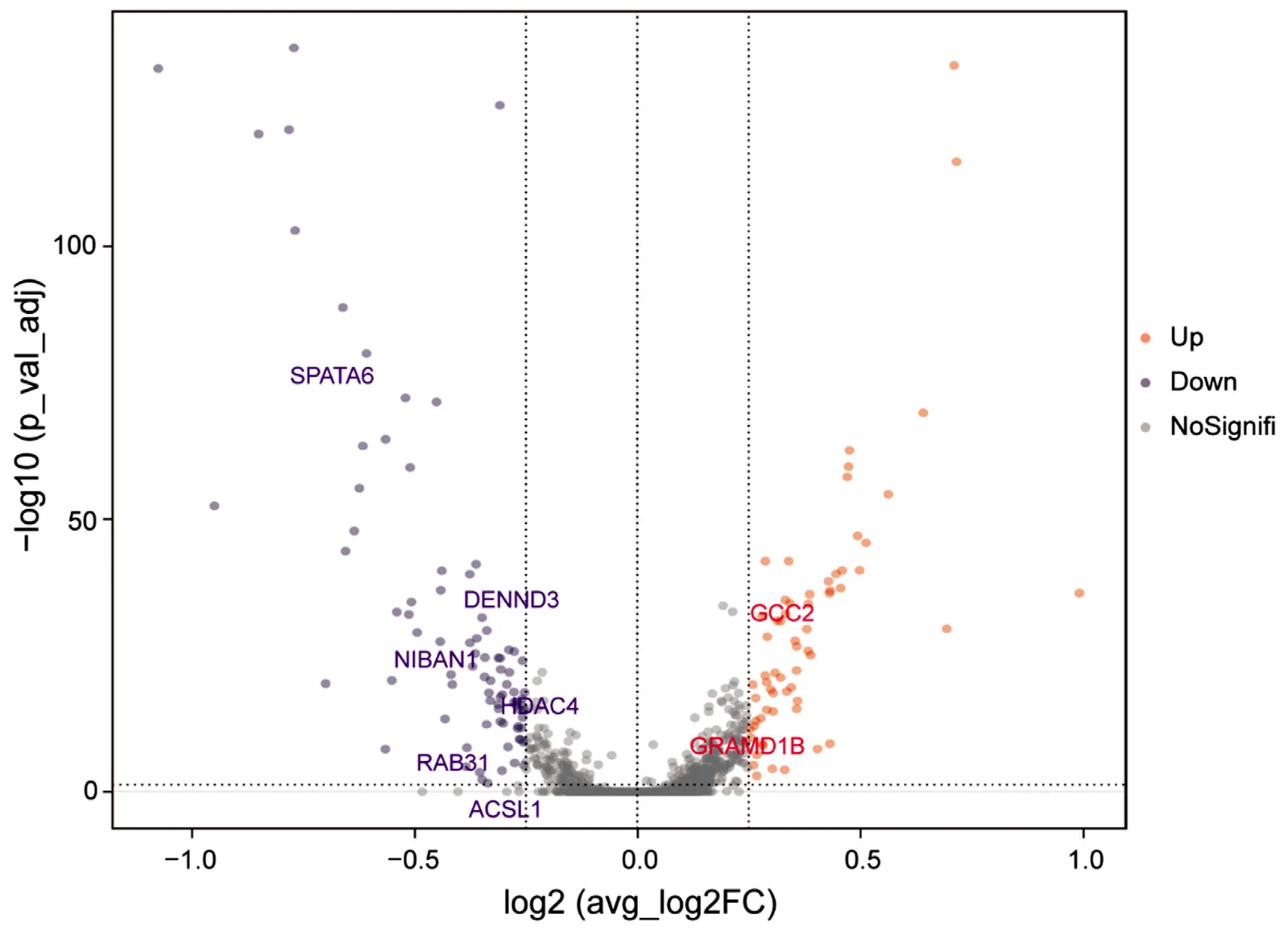
Volcano plot showing the differentially expressed genes in PVMs. Red and purple dots represent upregulated and downregulated genes, respectively.

To identify DEGs in PVMs between the disease and control groups, PVM data from both groups were extracted. A total of 149 DEGs were identified, as depicted in the volcano plot (Fig. **1**). These genes served as the gene set for subsequent analyses.

Enrichment analyses were performed to explore the regulatory relationships of these DEGs with various cellular functions. The results revealed significant enrichment in several functions and pathways, primarily those associated with immune and metabolic processes, including cellular response to organonitrogen compounds, GTPase regulator activity, cell activation, insulin receptor recycling, inflammatory response, macrophage activation, and positive regulation of the catabolic processes (Fig. **S3**). 

### 3.2 MR Analysis Identified Eight Genes among the DEGs in PVMs Associated with AD, one of Which was a Ferroptosis-related Gene

**Fig. (2):**
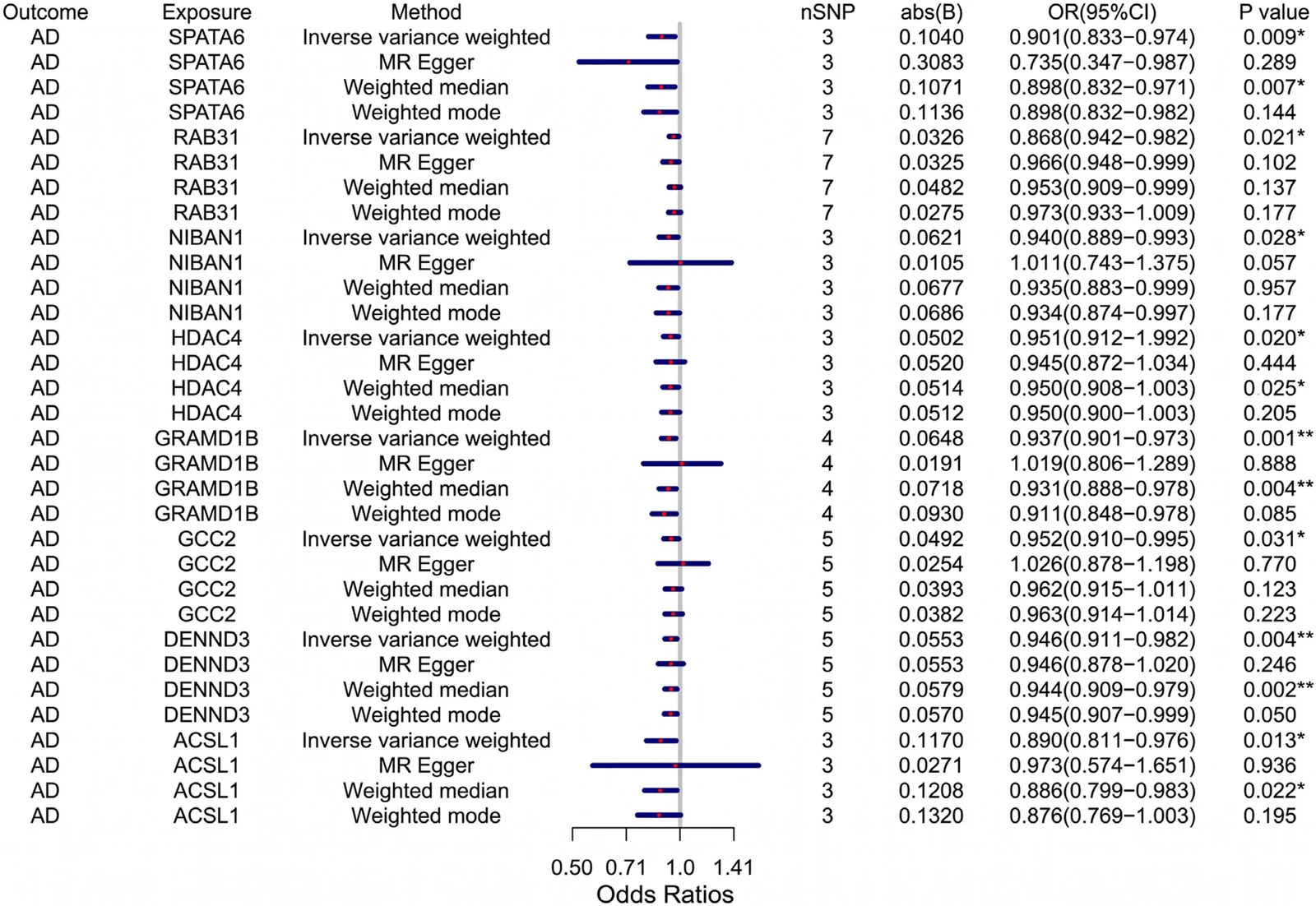
Forest plot depicting MR analyses of associations between the eight key genes and AD risk. *P < 0.05; **P < 0.005.

To pinpoint key AD-related genes among the identified DEGs, MR analysis was performed. The 149 DEGs were designated as exposure factors, while the summary statistics dataset, comprising 85,934 samples related to AD (46,828 controls and 39,106 cases), was used as the outcome data. Through MR analysis, causal relationships were identified for eight genes linked to the positive outcome of eQTL (Fig. **2**). Further analysis revealed significant inverse correlations between these genes and AD: *SPATA6*, *RAB31*, *NIBAN1*, *HDAC4*, *GRAMD1B*, *GCC2*, *DENND3*, and *ACSL1* (all IVW *P* < 0.05). By consulting the FerrDb V2 database, which classifies genes as drivers, suppressors, or markers of ferroptosis, we found that only *ACSL1* was identified as an FRG and belonged to the driver gene category.

**Fig. (3):**
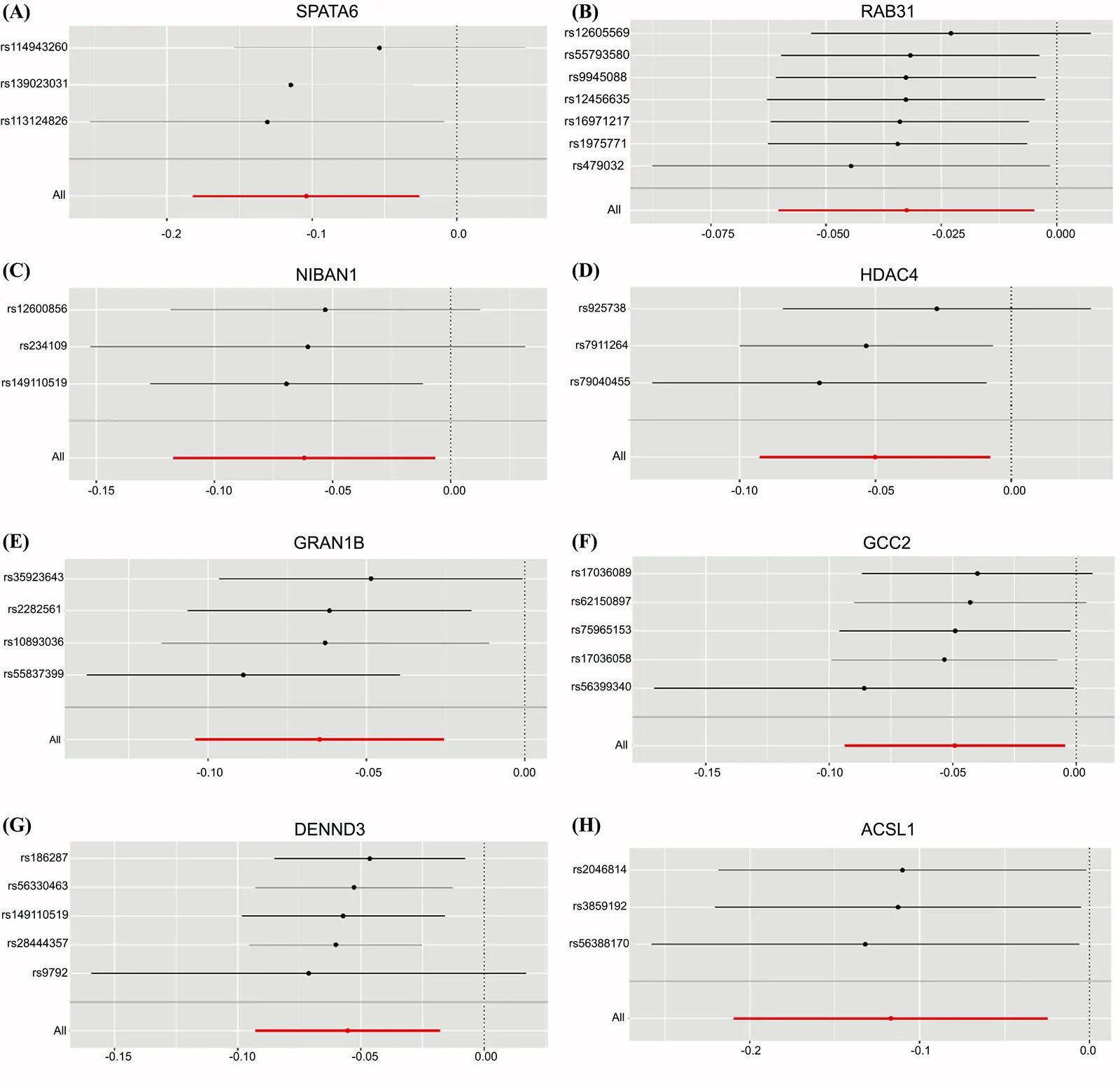
(**A**-**H**) Leave-one-out plots for assessing the robustness of causal associations between key genes and AD.

To ensure the robustness of these results, we performed a sensitivity analysis using leave-one-out analysis to examine the causal relationships of these eight genes. The findings demonstrated that removing any individual SNP did not significantly alter the overall estimates, confirming the stability of these causal associations (Figs. **3A**-**H**). Consequently, these eight genes were considered key genes for further analysis.

### 3.3 Immune Cell Infiltration Analysis

**Fig. (4):**
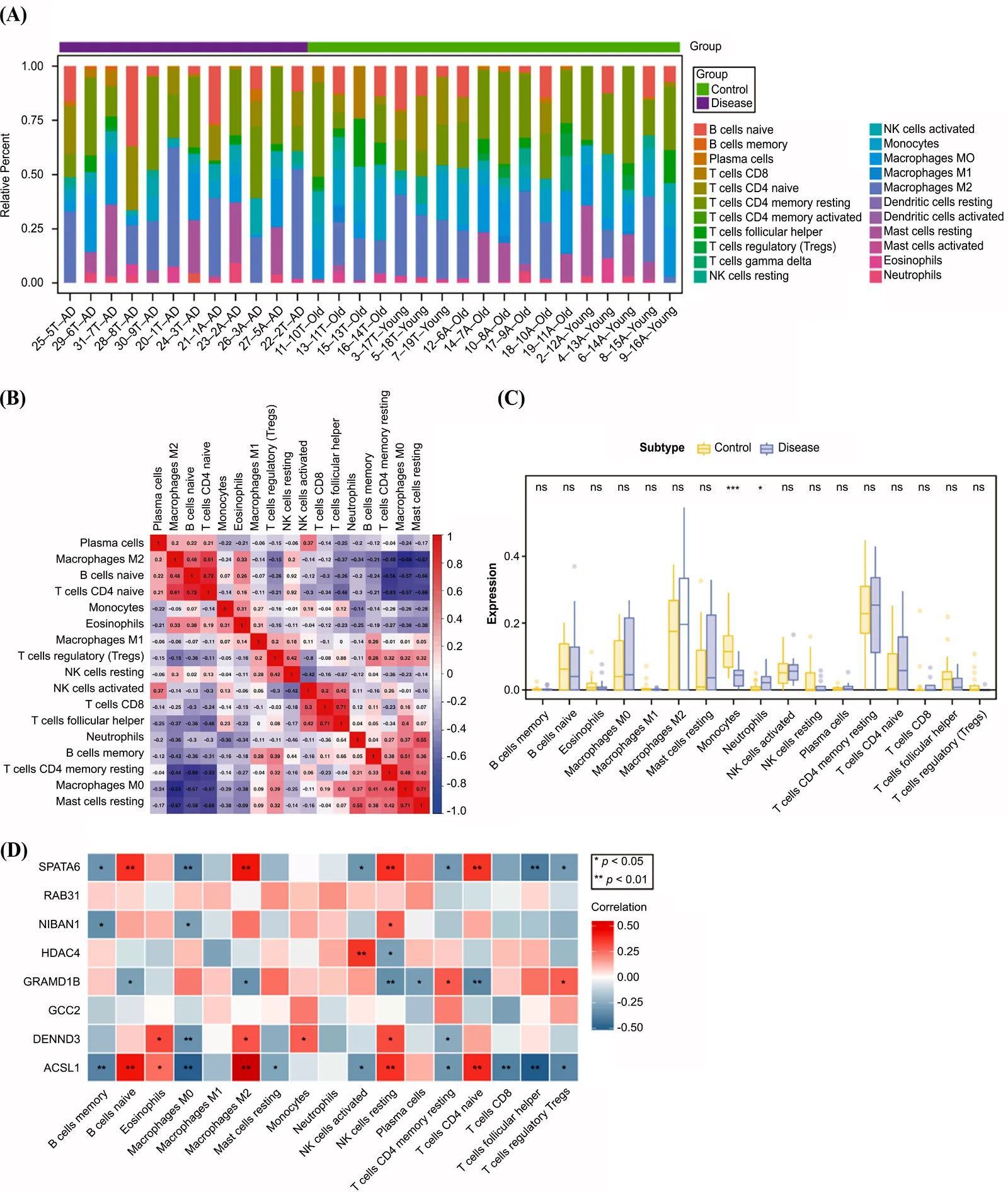
Assessment and visualization of immune cell infiltration. (**A**) Proportion of immune cell populations in the control and disease groups. (**B**) Heatmap showing correlations among immune cell types. (**C**) Boxplots depicting the abundance of various immune cell types between disease and control groups. (**D**) Correlations between key genes and immune cells. *P < 0.05; **P < 0.01; ***P < 0.001.

Previous studies have highlighted the crucial role of immune system dysfunction in AD pathogenesis. To investigate the role of PVMs, specialized innate immune cells in the brain, in the pathogenesis of AD, we explored the relationship between key genes and ICI in the AD dataset. We assessed the infiltration of 22 immune cell types in both groups and analyzed their correlations (Figs. **4A** and **B**). A comparative analysis between disease and control groups identified significant differences in monocyte and neutrophil infiltration (Fig. **4C**), suggesting a robust association between immune system alterations and AD pathogenesis.

To further explore the mechanisms by which key genes may influence AD progression, we analyzed their correlations with immune cell types. Several notable correlations were observed, with some key genes exhibiting significantly correlated relationships with multiple immune cell types. For instance, the ferroptosis-related gene *ACSL1* exhibited strong positive correlations with naïve B cells, M2 macrophages, resting NK cells, and naïve CD4 T cells, while showing significant negative correlations with memory B cells, M0 macrophages, CD8 T cells, and follicular helper T cells. Notably, *SPATA6* showed very similar performance to *ACSL1*. Conversely, both *RAB31* and GCC2 showed no significant association with immune cells, whereas *HDAC4* correlated positively only with NK cells. *DENND3* was the only gene significantly correlated with monocytes, whose infiltration differed considerably between the AD and control groups. Detailed results are shown in Fig. (**4D**).

We additionally utilized the TISIDB database to examine correlations between these key genes and various immune factors, including chemokines, immunosuppressive and immunostimulatory factors, and immune-associated receptors. Our analysis revealed that *ACSL1*, *DENND3*, *NIBAN1*, and *SPATA6* exhibit significant associations with multiple immunoinhibitors, Major Histocompatibility Complex (MHC) molecules, and immune-related receptors, revealing similar correlation patterns among these genes. In contrast, while *GCC2*, *HDAC4*, and *RAB31* exhibit similar expression profiles, they demonstrate minimal associations with these immune-related factors. Comprehensive correlation results are presented in Figs. (**5A**-**E**). These findings suggest that the identified key genes are strongly linked to ICI and play essential roles in modulating the immune microenvironment.

### 3.4 Expression Profiles of Key Genes in Diverse Cell Types and their Association with Different Functional Activities

 Our enrichment analysis indicated that DEGs in PVMs were predominantly implicated in immune and metabolic regulatory processes. To further understand these key genes and clarify potential interactions between immunometabolism and ferroptosis in the pathogenesis of AD, we profiled their expression across multiple brain-cell populations and examined their associations with a spectrum of functional activities, especially those related to immunometabolism.

 Our results revealed that these genes were predominantly expressed in PVMs, though some were also found in other cell types. Detailed cell-type-specific expression patterns are illustrated in Fig. (**S4**) and Fig. (**6A**). To determine their functional significance, we employed the AUCell function to assess gene enrichment levels across immune-related and metabolism-related, and other biological pathways. Pathway-specific activity differences were visualized with bubble charts. The eight key genes displayed significant associations with multiple immune and metabolism pathways. Specifically, *ACSL1* exhibited strong associations with several pathways, including the IL-6/JAK/STAT3 signaling pathway, the interferon-γ response, TGF-β signaling, Wnt/β-catenin signaling, bile acid metabolism, and cholesterol homeostasis. *SPATA6*, *RAB31*, and *NIBAN1* were positively correlated with cells exhibiting high Notch-signaling activity, whereas *GRAMD1B* and *DENND3* were negatively correlated with Cholesterol homeostasis. Beyond immune and metabolic processes, several genes were identified as being linked to additional pathways. For instance, *NIBAN1* was associated with apoptosis, and genes such as *SPATA6*, *RAB31*, *HDAC4*, and *ACSL1* showed significant correlations with angiogenesis. Fig. (**6B**) depicts these correlations in great detail. To gain a more comprehensive understanding of the function of these key genes, we also performed Gene Set Enrichment Analysis (GSEA) on the key genes in AD samples. The detailed results are presented in Fig. (**S5**).

### 3.5 Transcriptional Regulation Analysis of Key Genes

 To establish a foundation for future therapeutic interventions, we further predicted the TFs regulating these genes by annotating motifs enriched by the key genes based on their sequence similarities. The motif with the highest NES was cisbp_M1186, predominantly associated with *ACSL1*, *GRAMD1B*, and *HDAC4*. Additionally, some key genes were found to be regulated by common TFs. For instance, *ACSL1*, *DENND3*, *HDAC4*, and *SPATA6* were all regulated by CEBPD, whereas *DENND3*, *HDAC4*, and *SPATA6* were jointly regulated by the SP family members SP1, SP2, SP3, and SP4. Comprehensive information on enriched motifs and corresponding TFs is provided in Figs. (**7A** and **B**).

## 4 DISCUSSIONS

 AD is the primary cause of cognitive impairment in elderly patients. Demographic shifts, combined with the lack of effective treatments, suggest that the prevalence of AD will dramatically increase in the coming decades, highlighting the urgent necessity for disease-modifying therapies [46]. In this study, we identified eight genes in PVMs that were significantly negatively correlated with AD, including one recognized ferroptosis-related gene. By analyzing the relationships between these genes and both immune cell infiltration and various signaling pathways, we identified notable correlations with specific immune cell subsets and signaling pathways. These findings suggest that PVMs may contribute to AD pathology through mechanisms involving the crosstalk between immunometabolism and ferroptosis. Additionally, we predicted several TFs that regulate these genes, highlighting potential molecular targets for therapeutic intervention.

 We first applied scRNA-seq to characterize gene expression profiles of PVMs from patients with AD and healthy controls. This analysis identified 149 DEGs between these two groups, suggesting a potential involvement of PVMs in the pathogenesis of AD. Subsequently, enrichment analysis was performed to clarify the functional roles and biological pathways of these DEGs, revealing their predominant involvement in immune responses and metabolic regulation. These findings are consistent with the established roles of PVMs reported in previous studies [12, 13].

 To identify key AD-related genes among the DEGs and propose potential therapeutic targets, we employed MR analysis, a robust bioinformatic approach for examining large-scale genomic data and pinpointing genetic signatures associated with diseases. Through this analysis, we identified eight genes exhibiting significant inverse correlations with AD, indicating their potential protective roles against the disease. Among these eight genes, *ACSL1* is cataloged in the ferroptosis-related gene database FerrDb V2, confirming its significant role in ferroptosis [47].

*ACSL1* (Acyl-CoA Synthetase Long-chain family member 1) participates in fatty acid metabolism by catalyzing the conversion of long-chain fatty acids into acyl-CoA esters, a critical step in lipid biosynthesis and β-oxidation, and is well-established as a ferroptosis regulator [48]. However, studies indicate that *ACSL1* has a dual role in regulating ferroptosis, exhibiting both pro-ferroptotic and anti-ferroptotic functions, depending on the cellular context. In a study investigating diabetic nephropathy, reduced expression of protein arginine methyltransferase 6 (PRMT6) induced lipid peroxidation by upregulating *ACSL1*, contributing to ferroptosis [49]. Similarly, in acute myeloid leukemia, inhibition of protein arginine methyltransferase 1 (PRMT1) promoted sensitivity to ferroptosis *via ACSL1* upregulation [50]. This pro-ferroptotic role was further corroborated in head and neck squamous cell carcinoma, where transcriptomic analyses identified *ACSL1* as a central gene associated with ferroptosis and worse overall survival [51]. The pro-ferroptotic functions of *ACSL1* appear to be mediated through several molecular pathways. A primary mechanism is that *ACSL1* facilitates the incorporation of PolyUnsaturated Fatty Acids (PUFAs) into membrane phospholipids, thereby increasing cellular susceptibility to lipid peroxidation—the hallmark biochemical feature of ferroptosis. Conversely, in ovarian cancer, *ACSL1* demonstrated anti-ferroptotic properties by enhancing antioxidant capacity and increasing ferroptosis resistance through the modulation of Ferroptosis Suppressor Protein 1 (FSP1) N-myristoylation and stability [52]. This contradiction highlights the context-dependent nature of ACSL1’s function, which tissue-specific factors and the surrounding metabolic environment may influence. 

Previous studies have shown that increased ACSL1 expression in microglia, the primary resident immune cells of the brain, leads to lipid droplet accumulation, suggesting that *ACSL1* may mediate the detrimental effects of lipid metabolism in microglia, potentially promoting ferroptosis and contributing to AD progression [53]. In contrast, our current research indicates that elevated *ACSL1* in the PVMs of AD patients plays a protective role, implying that *ACSL1* may exert anti-ferroptotic effects. The paradoxical role of *ACSL1* in ferroptosis within microglia and PVMs may stem from their distinct microenvironmental adaptations and metabolic programming. In microglia, which are primarily located in the brain parenchyma, *ACSL1* may predominantly direct PUFAs into membrane phospholipids, increasing susceptibility to ferroptosis. In contrast, PVMs, situated around blood vessels and continuously exposed to blood-derived iron and lipids, may have evolved to couple *ACSL1* activity with enhanced antioxidant defense mechanisms and specialized lipid mediator production, thereby counteracting the ferroptosis process. However, the specific mechanisms necessitated further experimental verification. Moreover, to clarify the potential roles and mechanisms of these genes in the crosstalk between immunometabolism and ferroptosis, we further analyzed their correlations with a range of immunometabolic pathways. We found that *ACSL1* displayed strong associations with several pathways already implicated in ferroptosis, including IL-6/JAK/STAT3 signaling, the interferon-γ response, TGF-β signaling, Wnt/β-catenin signaling, bile acid metabolism, and cholesterol homeostasis [54-57]. These results suggest that ACSL1 in PVMs may mediate the intersection of immunometabolism and ferroptosis through these pathways, thereby contributing to AD pathogenesis. Further investigation is needed to validate these assumptions.

*HDAC4* is a member of the class IIa histone deacetylases (HDACs) and has emerged as a key epigenetic regulator, modulating the transcription of genes involved in synaptic plasticity, neuronal survival, and neurodevelopment by interacting with various proteins, including transcription factors and signal transduction molecules [58]. Due to its central role in modulating synaptic plasticity, the role of HDAC4 in cognitive function has been extensively explored [58]. Aberrant expression of *HDAC4* was found to be correlated with cognitive impairment in neurodegenerative diseases and mental disorders, suggesting it as a potential therapeutic target for alleviating cognitive defects in such diseases [59]. Previous studies have shown that HDAC4 is involved in linking metabolic reprogramming to ferroptosis resistance. Lipotoxicity increased *HDAC4* expression, which in turn reduced the expression of metabolic genes and impaired oxidative metabolism in skeletal muscle. This metabolic reprogramming was associated with impaired apoptosis and ferroptosis responses [60]. The role of *HDAC4* in inhibiting ferroptosis through metabolic reprogramming presents an intriguing parallel to AD pathophysiology. AD is characterized by metabolic dysregulation, including altered glucose metabolism and mitochondrial dysfunction [61]. *HDAC4*’s ability to modulate metabolic pathways and suppress ferroptosis suggests it may contribute to neuronal survival in AD. The convergence of *HDAC4*’s roles in metabolic regulation, ferroptosis inhibition, and neuronal function suggests it may represent a critical node in AD pathophysiology, connecting metabolic alterations to neurodegeneration through ferroptosis-related mechanisms.

 The primary role of *GRAMD1B* (GRAM domain containing 1B) is to maintain cholesterol distribution and lipid homeostasis within the cell. *GRAMD1B* facilitates the transport of excess cholesterol from the trans-Golgi network to the endoplasmic reticulum. Cells deficient in *GRAMD1B* accumulate cholesterol in the plasma membrane [62], a process intimately linked to ferroptosis sensitivity. Disruption of lipid homeostasis is a well-established feature of AD, with alterations in membrane lipid composition affecting amyloid precursor protein processing and tau phosphorylation [63]. *GRAMD1B*'s role in cholesterol transport between organelle membranes may influence membrane lipid composition, thereby impacting ferroptosis sensitivity in neurons. Although there is no direct evidence for *GRAMD1B*’s involvement in ferroptosis, the potential interplay between *GRAMD1B*-mediated lipid transport, membrane lipid composition, and ferroptosis susceptibility represents a novel avenue for understanding AD pathophysiology. Targeting *GRAMD1B* to modulate lipid homeostasis could potentially influence ferroptosis sensitivity in neurons, offering a new therapeutic approach for AD. Additionally, our analysis demonstrated a significant positive correlation between *GRAMD1B* expression and PI3K/AKT/mTOR signaling, suggesting that this pathway may mediate the crosstalk between immunometabolism and ferroptosis.

Earlier research revealed that *RAB31*, a member of the RAS oncogene family, plays a pivotal role in controlling exosome biogenesis through an Endosomal Sorting Complex Required for Transport (ESCRT)-independent process [64]. AD development is closely associated with the spread of pathogenic Aβ and tau proteins, an event increasingly recognized to engage exosomes [65, 66]. There is also evidence that suggests RAB31 participates in packaging Aβ into exosomes through an ESCRT-independent process, facilitating the dissemination of AD pathogenesis [67]. Other scientists have similarly reported *RAB31* as a significant gene that plays a role in AD pathogenesis [68]. Cumulatively, these studies indicate that *RAB31* could be an intervention target for AD. We also established that *RAB31* was involved in several signaling pathways, including Notch signaling, heme metabolism, and TGF-β signaling. These pathways could provide valuable insights into the role of RAB31 in disease development.

*DENND3* functions as a guanine nucleotide exchange factor for Rab GTPases, inducing autophagosome formation and trafficking by activating Rab12, which is essential for autophagy regulation [69]. Recently, a study employed diverse bioinformatics tools and machine-learning approaches to establish *DENND3* as a strong biomarker for diagnosing Moyamoya disease [70]. Based on our research, we also identified *DENND3* as a significant gene involved in AD, suggesting that it may play a role in the disease's pathology. Nonetheless, additional verification is still needed to establish whether *DENND3*’s function in AD is related to its role in regulating autophagy. It is worth noting that our immune cell infiltration analysis showed significant differences in the abundance of monocytes and neutrophils between the AD and control groups. Among the eight candidate genes, only *DENND3* showed a significant association with monocyte levels, suggesting a potential mechanistic link.

While both *RAB31* and *DENND3* have been implicated in AD, no existing studies have yet confirmed their relationship with ferroptosis. Given that both are involved in vesicular trafficking, which is essential for the transport of iron, lipids, and antioxidants—all critical components in ferroptosis regulation—the potential intersection of vesicular trafficking defects with ferroptosis susceptibility represents a novel perspective on AD pathophysiology. Previous studies have shown that *DENND3*, when phosphorylated by ULK1, activates Rab12, a small GTPase that, upon activation, directs the transferrin receptor 1 (TfR1) to lysosomes for degradation [69, 71, 72]. This mechanism reduces iron import and dynamically modulates the labile iron pool that drives ferroptosis. Additionally, *RAB31* mediates an ESCRT-independent exosome pathway that potentially packages ferritin, GPX4, and GSH into intraluminal vesicles for secretion [64]. By exporting iron-laden or antioxidant cargo, *RAB31* may act as an intrinsic buffer that dissipates labile-iron stress while simultaneously modulating the redox tone of neighbouring neurovascular elements. However, the hypothesis that alterations in *RAB31* and *DENND3* could disrupt the transport of essential ferroptosis regulators, such as glutathione, iron, and lipids, thereby altering neuronal susceptibility to ferroptotic cell death in AD, warrants further investigation.

The other three key genes, *GCC2*, *SPATA6*, and *NIBAN1*, have not been extensively studied in the context of the AD condition. However, some studies suggest that *SPATA6* and *NIBAN1* may be involved in cellular stress responses [73, 74]. Given that the pathological mechanisms of AD involve stress, such as protein misfolding due to endoplasmic reticulum stress [75, 76], *SPATA6* and *NIBAN1* could potentially influence AD pathogenesis through their roles in managing cellular stress. Moreover, direct evidence linking these genes to ferroptosis is limited; however, their known cellular functions suggest potential involvement. Specifically, *NIBAN1*'s participation in stress-response mechanisms might influence cellular resilience to ferroptotic stimuli, while *SPATA6*'s cytoskeletal functions might influence cellular structural changes during ferroptosis. Considering that both cytoskeletal stability and cellular stress responses are disrupted in AD, these genes may influence neuronal susceptibility to ferroptosis within the disease context.

Additionally, our results showed that *NIBAN1* was preferentially expressed in cells with heightened immune-related signaling activity, such as the IL-6/JAK/STAT3 signaling pathway and the interferon-γ response. In contrast, both *NIBAN1* and *SPATA6* were enriched in cells displaying high Notch signaling activity. These results highlight promising directions for future research, and additional studies are needed to validate our observations and clarify the roles of *NIBAN1* and *SPATA6* in ferroptosis regulation and AD pathophysiology. Notably, beyond their immune and metabolic functions, our data suggest robust associations of *SPATA6*, *RAB31*, *HDAC4*, and *ACSL1* with angiogenesis. Future research could explore the roles of these genes in angiogenesis to deepen our understanding of the relationship between PVMs and adverse effects, such as microhemorrhages and vasogenic edema, associated with the newly approved amyloid-targeting therapies.

We also predicted the TFs regulating these key genes to provide insights for future therapeutic interventions. Our analysis revealed that multiple key genes shared regulations by common TFs. For example, previous studies have shown that TFs Sp1 and Sp3 directly bind to GC-rich sequences within the *HDAC4* promoter, facilitating its transcription [77]. We discovered that, in addition to Sp1 and Sp3, Sp2 and Sp4 also contribute to regulating *HDAC4* expression. Notably, beyond *HDAC4*, Sp1, Sp2, Sp3, and Sp4 were also identified as TFs for *DENND3* and *SPATA6*, highlighting a potential therapeutic approach for addressing AD pathology by modulating these shared TFs.

Despite its strengths, the present study has several limitations. As our GWAS samples primarily comprised individuals from European ancestry, the findings represent only a small segment of the broader population. Expanding sample sizes and increasing population diversity may provide additional insights. Additionally, animal experiments or well-conducted Randomized Controlled Trials (RCTs) are warranted to confirm the results further. Therefore, these limitations should be carefully considered when interpreting the findings of this study.

## CONCLUSION

In summary, we identified eight key genes causally associated with AD through a combination of scRNA-seq and MR analysis. Our research not only advances the understanding of PVMs’ roles in AD but also provides a robust foundation for developing novel therapeutic strategies. By targeting these critical genes and pathways, future research may lead to more effective treatments that significantly improve the prognosis for individuals affected by this devastating neurodegenerative disease.

## Figures and Tables

**Fig. (5):**
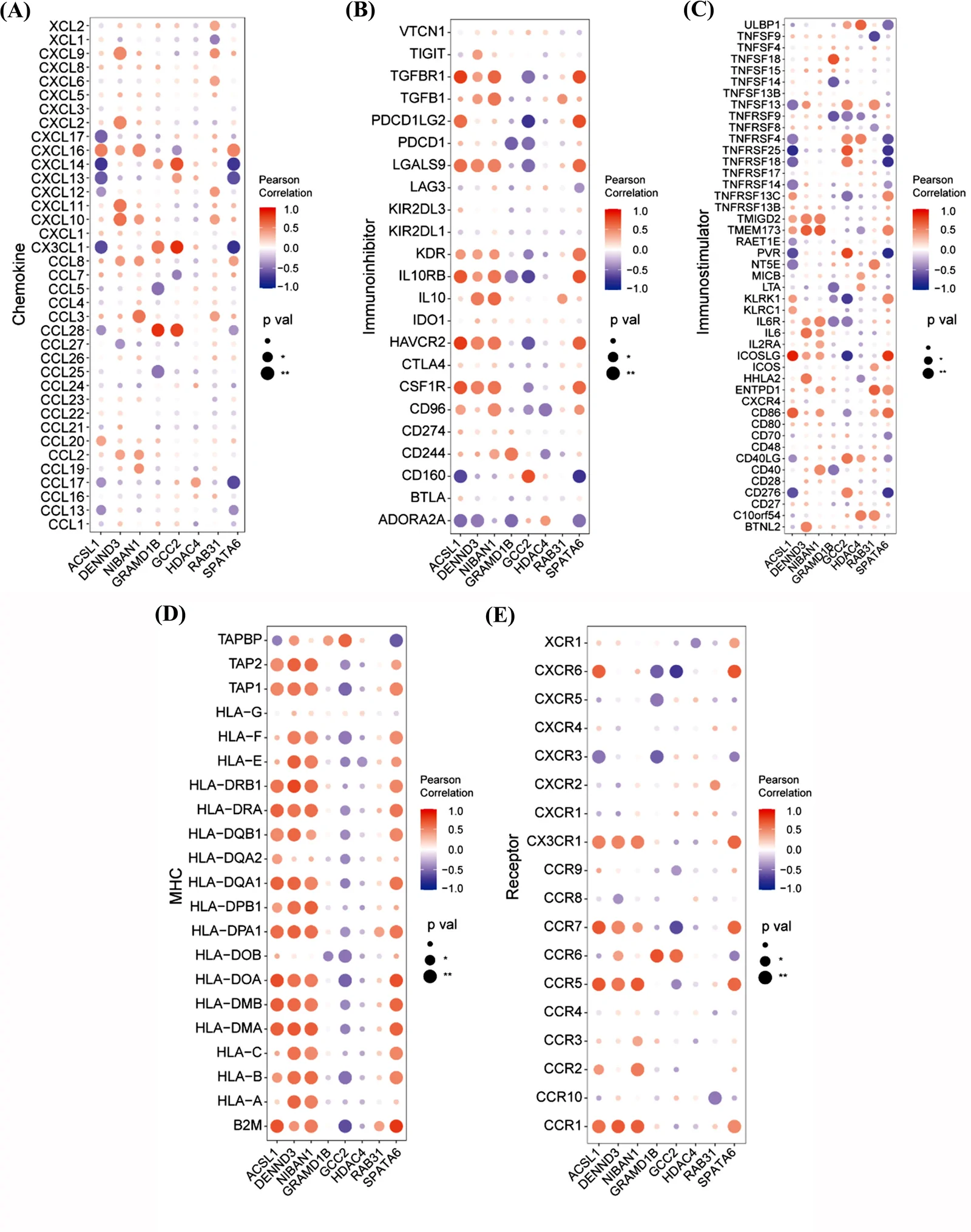
Correlation of key genes with (**A**) chemokines, (**B**) immunoinhibitors, (**C**) immunostimulators, (**D**) major histocompatibility complex (MHC), and (**E**) receptors.

**Fig. (6):**
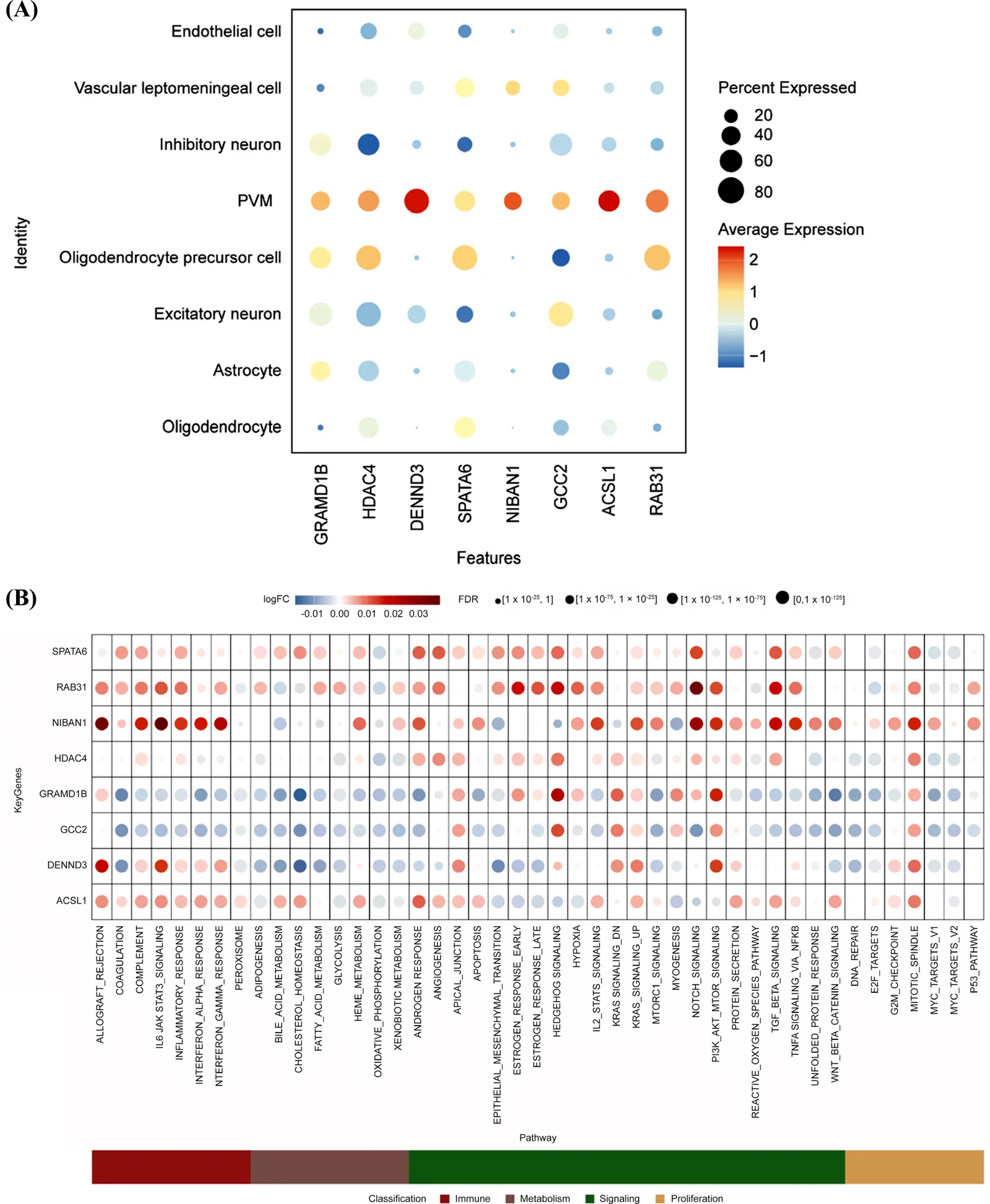
Expression profiles of key genes and their correlation with different functional activities in single-cell data. (**A**) Expression levels of key genes in each cell type from AD samples. (**B**) Bubble chart presenting activity variances of key genes across different functional activities.

**Fig. (7):**
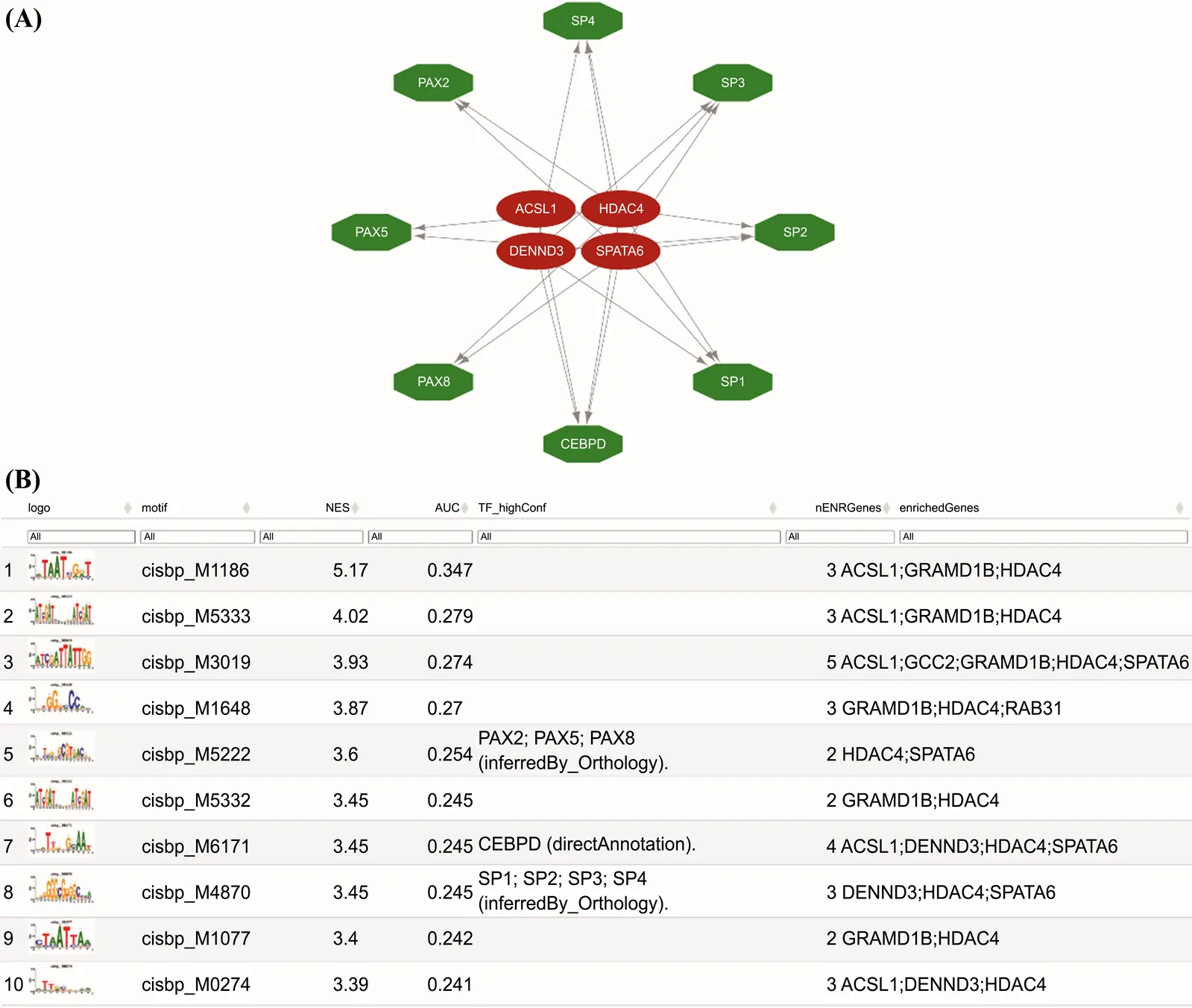
Transcriptional regulation analysis of key genes. (**A**) Predicted TFs of the key genes. (**B**) Motifs enriched for the key genes.

## Data Availability

This study analyzed publicly available datasets. These data can be found here: the GEO database (https://www.ncbi.nlm.nih.gov/geo/info/datasets.html), the eQTLGen consortium's database (https://eqtlgen.org/), and the EBI database (https://gwas.mrcieu.ac.uk/).

## LIST OF ABBREVIATIONS

AD: Alzheimer's Disease
AUC: Area under the Curve
Aβ: Amyloid Beta Protein
CNS: Central Nervous System
DEGs: Differentially Expressed Genes
eQTL: expression of Quantitative Trait Loci
ESCRT: Endosomal Sorting Complex Required for Transport
GO: Gene Ontology
GWAS: Genome-wide Association Studies
HDACs: Histone Deacetylases
ICI: Immune Cell Infiltration
IVs: Instrumental Variables
IVW: Inverse Variance Weighted
KEGG: Kyoto Encyclopedia of Genes and Genomes
LD: Linkage Disequilibrium
MAD: Median Absolute Deviation
MR: Mendelian Randomization
NES: Normalized Enrichment Score
OR: Odds Ratio
PCA: Principal Component Analysis
PVMs: Perivascular Macrophages
SNPs: Single-nucleotide Polymorphisms
TFs: Transcription Factors
UMAP: Uniform Manifold Approximation and Projection
UMI: Unique Molecular Identifier
